# DXA evaluation of bone fragility 2 years after bariatric surgery in patients with obesity

**DOI:** 10.1016/j.bonr.2024.101782

**Published:** 2024-06-22

**Authors:** Marine Fauny, Marion Halin, Edem Allado, Laurent Brunaud, Claire Nomine-Criqui, Eliane Albuisson, Isabelle Chary-Valckenaere, Didier Quilliot, Damien Loeuille

**Affiliations:** a Department of Rheumatology, University Hospital, Nancy, France; b Department of Rheumatology, Saint Charles Hospital, Toul, France; c CHRU-Nancy, University Center of Sports Medicine and Adapted Physical Activity, F-54000 Nancy, France; d Université de Lorraine, DevAH, F-54000 Nancy, France; e Unité Multidisciplinaire de la Chirurgie de l'obésité (UMCO), University Hospital, Nancy, France; f Inserm UMRS 1256 N-GERE (Nutrition-Genetics-Environmental Risks) - University de Lorraine, Faculty of Medicine, Nancy, France; g Department of Gastrointestinal, Visceral, Metabolic, and Cancer Surgery (CVMC), University Hospital, Nancy, France; h Université de Lorraine, CNRS, IECL, F-54000 Nancy, France; i CHRU-Nancy, DRCI, Département MPI, Unité de méthodologie, Data management et statistiques UMDS, F-54000 Nancy, France; j Ingénierie Moléculaire et Physiopathologie Articulaire (IMoPA). UMR 7365 CNRS –University of Lorraine, France; k Department of Endocrinology Diabetology and Nutrition, University Hospital, Nancy, France

**Keywords:** Obesity, Bariatric surgery, Bone mineral density (BMD), Osteoporosis, Dual X-ray absorptiometry (DXA)

## Abstract

**Purpose:**

The primary objective was to evaluate bone fragility on dual X-ray absorptiometry (DXA) in patients with obesity before and 2 years after bariatric surgery. The secondary objective was to identify risk factors for the development of a bone mineral density ≤ −2 SD at 2 years.

**Methods:**

This descriptive study included patients with obesity who underwent DXA before and 2 years (±6 months) after bariatric surgery. The BMD and the T-score were assessed at the lumbar spine, femoral neck and total hip. Data on body composition on DXA were also collected. The diagnosis of osteoporosis was retained for a T-score ≤ − 2.5 SD at any measured location. Osteopenia, or low bone mass, was defined by −2.5 SD < T-score ≤ −1 SD.

**Results:**

Among the 675 included patients, 77.8 % were women, with a mean age of 49.5 years (±11.1). After bariatric surgery, there were significantly more patients with osteoporosis: 3.6 % vs. 0.9 % (*p* = 0.0001). Multivariate analysis revealed that the risk factors for developing a bone mineral density ≤ −2 SD 2 years after bariatric surgery in patients with normal BMD before surgery were age and lower lean and fat mass before the surgery (OR = 1.07, 95%CI = [1.03–1.12], OR = 0.83, 95%CI = [0.77–0.91], OR = 1.08, 95%CI = [1.02–1.15], respectively).

**Conclusion:**

There was a significantly higher prevalence of osteoporosis and low bone mass 2 years after bariatric surgery. Older age and lower lean and fat mass at baseline were risk factors for the development of a BMD ≤ -2SD at 2 years.

## Highlights

• Higher prevalence of osteoporosis and low bone mass after bariatric surgery
• Bone screening before and after bariatric surgery to diagnose osteoporosis
• Especially in older patients with a lower lean and fat mass at baseline

## 1 Introduction

Obesity and osteoporosis have become major global health problems due to their increasing prevalence (Sarafrazi, 2021; Apovian, 2016). The interaction between these two diseases is complex and not fully understood.

Osteoporosis is a disease characterized by a decrease in bone mass and deterioration of bone microarchitecture leading to fragility fractures (Lane et al., 2000). The gold standard for osteoporosis diagnosis is dual X-ray absorptiometry (DXA) (Dimai, 2017).

Obesity is defined by a BMI ≥ 30 kg/m^2^ in Caucasian population (WHO, 2000). Its prevalence is increasing worldwide (Apovian, 2016). Bariatric surgery is recognized as a highly effective therapy for obesity, allowing weight loss, a reduction in comorbidities and mortality, and an improvement in quality of life (Borisenko et al., 2015; Lupoli et al., 2017). In the literature, obesity seems to be a protective factor for bone (Murray et al., 2017; Lespessailles et al., 2019a), but some studies have shown a higher risk of fracture in patients with obesity (Gonnelli et al., 2014; Tencerova et al., 2019), probably due to an alteration of the bone architecture, with increased bone marrow adipose tissue (Ambrosi et al., 2017; Boskey and Imbert, 2017; Naveiras et al., 2009). According to many studies, malabsorptive procedures lead to a decrease in bone mineral density and sometimes an increased risk of fragility fractures (Geoffroy et al., 2019; Axelsson et al., 2018; Lespessailles et al., 2019b; Ko et al., 2016; Lu et al., 2015). A reduction in bone mineral density (BMD) and an increase of bone turnover markers was previously described (Hofsø et al., 2020). However, the kinetics of bone loss and its physiopathology are unclear.

In French recommendation, it is recommended to perform routinely an initial assessment of fracture risk ideally before the first bariatric surgery procedure in the case of RYGB and biliopancreatic diversion, and in patients at high risk of fracture, regardless of age, and in all menopausal women and all men ≥50 years old, regardless of the type of bariatric surgical procedure. Anti-osteoporosis treatment is indicated for menopausal women and men ≥50 years old with no history of fracture and a T-score ≤ −2 (Paccou et al., 2022a).

The primary objective of this study was to evaluate bone fragility on DXA in patients with obesity before and 2 years after bariatric surgery. The secondary objective was to identify risk factors for developing abnormal BMD ≤ −2 SD at 2 years.

## 2 Materials and methods

### 2.1 Population

This descriptive study included patients with obesity who underwent bariatric surgery in our specialized obesity center between January 2014 and December 2019. To be included, patients had to have undergone DXA before and two years (±6 months) after bariatric surgery. All exams had to be performed at the same center during routine pre- and postoperative follow-up visits. The exclusion criteria were gastric banding and ring ablation. Demographic and anthropometric data (age, sex, smoking and alcohol consumption habits, body height and weight to calculate BMI) and comorbidities such as diabetes and cardiovascular risk factors (dyslipidemia, hypertension) were collected from complete medical records. To evaluate osteoporosis, clinical risk factors (sex, age, smoking and alcohol consumption habits) and vitamin D status were also collected (vitamin D deficiency was defined by a level below 30 ng/mL). Vitamin D evaluation was performed in the same laboratory with chemiluminescence immunoassay (Diasorin).

### 2.2 DXA evaluation

Any available pre- and postoperative DXA was used. The preoperative DXA closest to the date of surgery and postoperative DXA performed between 18 and 30 months after surgery (2 years ±6 months) were retained. All DXA was performed on a Lunar Prodigy densitometer (Advance PA +301,010, Encore, version 14.10.022; Madison, WI, 53718, USA). The BMD and the T-score were assessed for each patient at the lumbar spine, femoral neck and total hip. The diagnosis of osteoporosis was retained for a T-score ≤ − 2.5 SD at any measured location. Osteopenia, or low bone mass, was defined by −2.5 SD < T-score ≤ −1 SD (*World Health Organ. Tech. Rep. Ser.*, 1994). Data on lean and fat mass and their distribution (arms, legs, trunk, android vs. gynoid fat and lean mass and visceral fat mass) were also collected from DXA whole body examination (limbs included).

### 2.3 Ethics approval

All of the data used were obtained from medical records. No examinations were performed to determine if the patients met the inclusion criteria. This study is registered with the Information Technology and Freedoms Commission for our University Hospital and on Clinicaltrials.gov (file number: 2019PI216) and was designed in accordance with the general ethical principles outlined in the Declaration of Helsinki. The protocol of this study was approved by the Information Technology and Freedoms Commission for our University Hospital. All patients gave their consent for the use of their medical data from the time period they received medical care at the University Hospital.

### 2.4 Statistical analysis

Both descriptive and comparative analyses were conducted by accounting for the nature and distribution of the variables. Qualitative variables are described as frequencies and percentages; quantitative variables are reported as the mean ± SD or as the median and interquartile range (IQR). The Kolmogorov–Smirnov test showed that among the continuous demographic and clinical variables, only age, height, and weight followed a normal distribution.

For comparisons of the data before and two years after surgery, a paired Student's *t-*test and Chi-squared test were used for variables with a normal distribution, and the Wilcoxon signed rank test and Mann-Whitney *U* test were used for other continuous variables. For qualitative variables, the McNemar test was used, and logistic regression was performed to identify the variables significantly associated with the appearance of a T-score ≤ −2 SD. Significant results (univariate and multivariate analysis) are presented with their *p* values, odds ratio (OR) and 95 % confidence interval (95%CI). The significance level was set at 0.05 for the entire study. IBM SPSS Statistics v23 was used for data analysis.

## 3 Results

### 3.1 Population

In total, 675 patients underwent DXA before and two years (±6 months) after bariatric surgery. The characteristics of the patients are described in Table 1.

**Table 1:** Characteristics of the patients at baseline (*n* = 675).

Demographical data	
Age (years)	49.5 (11.1)
Sex (women)	525 (77.8)
Weight (kg)	124.9 (22.4)
Height (cm)	165.9 (9.0)
BMI (kg/m2)	45.3 [6.5]
Diabetes	207 (30.7)
Cardiovascular risk factors	406 (60.1)
Tobacco	258 (38.2)
Alcohol	24 (3.6)
Vitamin D deficiency	560 (83)


Type of surgery	
GBP	619 (91.7)
Sleeve gastrectomy	56 (8.3)


DXA	
Osteoporosis on at least one site	6 (0.9)
Low bone mass on at least on site	82 (12.1)
Femoral neck	
BMD (g/cm2)	1.093 [0.15]
T-score	0.85 [1.21]
Osteoporosis	2 (0.3)
Low bone mass	48 (7.1)
Hip	
BMD (g/cm2)	1.169 [0.15]
T-score	1.33 [1.17]
Osteoporosis	0 (0)
Low bone mass	17 (2.5)
Spine	
BMD (g/cm2)	1.268 [0.16]
T-score	0.81 [1.33]
Osteoporosis	6 (0.9)
Low bone mass	49 (7.3)
Body composition	
Lean mass (kg)	57.7 [11.0]
Fat mass (kg)	62.7 [14.0]
Lean mass/total weight (%)	33.6 [6.7]
Fat mass/total weight (%)	37.8 [8.0]
Android fat mass/gynoid fat mass	0.8 [0.4]
Appendicular/central fat mass	0.8 (0.2)
ALM/H^2^	16.9 [3.0]
ALM/W	0.2 [0.03]
VAT (g)	2760.4 [1385.2]

Data are presented as n (%) for dichotomous variables, the mean (SD) for continuous demographic variables with a normal distribution and the median [interquartile range] for those with a nonnormal distribution. The percentage was calculated based on the available data for each variable.

ALM/H^2^: appendicular lean mass adjusted to height; ALM/W: appendicular lean mass adjusted to body weight; BMD: bone mineral density; BMI: body mass index; DXA: dual-energy X-ray absorptiometry; GBP: gastric bypass; VAT: visceral adipose tissue. Osteoporosis was defined by a T-score ≤ −2.5 SD at any measured location, and low bone mass was defined by −2.5 SD < T-score ≤ −1 SD. Vitamin D deficiency was defined by a level below 30 ng/mL.

At baseline, before bariatric surgery, the mean age of the patients was 49.5 years (±11.1), 77.8 % were women, the mean weight was 124.9 kg (±22.4), and the mean BMI was 45.3 kg/m^2^ (±6.5); 30.7 % of patients had diabetes, and 60.1 % had cardiovascular risk factors. In total, 38.2 % of the patients used tobacco, and 3.6 % consumed alcohol; 83 % of the patients presented vitamin D deficiency.

### 3.2 Patient characteristics two years after surgery

Two years after bariatric surgery, the mean weight was 82.6 kg (±18.6), and the mean BMI was 29.9 kg/m^2^ (±5.7). The mean weight change was 42.3 kg (±15.1). The change in BMI was statistically significant (*p* = 0.0001).

There were more patients with osteoporosis or low bone mass (at the femoral neck, hip and spine): 6 patients (0.9 %) had osteoporosis in at least one site before surgery, and 24 (3.6 %) had osteoporosis two years after surgery. This difference was statistically significant (p = 0.0001). Their T-score and BMD were significantly lower after bariatric surgery (p = 0.0001 for each).

The patients' characteristics at 2 years and the comparison with baseline characteristics are described in Table 2.

**Table 2:** Comparison of the included patients' characteristics before and at 2 years after bariatric surgery (n = 675).

	Before	2 years after	p value
Demographical data			
Weight (kg)	124.9 (22.4)	82.6 (18.6)	**0.0001**
BMI (kg/m2)	45.3 [6.5]	29.9 [5.7]	**0.0001**
DXA			
Osteoporosis on at least one site	6 (0.9)	24 (3.6)	**0.0001**
Low bone mass on at least on site	82 (12.1)	172 (25.5)	**0.0001**
Femoral neck			
BMD (g/cm2)	1.093 [0.15]	0.999 [0.15]	**0.0001**
T-score	0.85 [1.21]	0.03 [0.15]	**0.0001**
Osteoporosis	2 (0.3)	11 (1.6)	**0.004**
Low bone mass	48 (7.1)	114 (16.9)	**0.0001**
Hip			
BMD (g/cm2)	1.169 [0.15]	1.046 [0.16]	**0.0001**
T-score	1.33 [1.17]	0.25 [1.23]	**0.0001**
Osteoporosis	0 (0)	7 (1.03)	**/**
Low bone mass	17 (2.5)	106 (15.7)	**0.0001**
Spine			
BMD (g/cm2)	1.268 [0.16]	1.217 [0.17]	**0.0001**
T-score	0.81 [1.33]	0.26 [1.42]	**0.0001**
Osteoporosis	6 (0.9)	17 (2.5)	**0.02**
Low bone mass	49 (7.3)	111 (16.4)	**0.0001**
Body composition			
Lean mass	57.7 [11.0]	49.8 [10.0]	**0.0001**
Lean mass/total weight (%)	33.6 [6.7]	61.0 [11.4]	**0.0001**
Fat mass	62.7 [14.0]	30.9 [12.6]	**0.0001**
Android fat mass/gynoid fat mass	0.8 [0.4]	1.32 [0.77]	**0.0001**
Appendicular/central fat mass	0.8 (0.2)	0.95 (0.3)	**0.0001**
ALM/H^2^	16.9 [3.0]	14.6 [3.3]	**0.0001**
ALM/W	0.2 [0.03]	0.29 [0.08]	**0.0001**
VAT (g)	2760.4 [1385.2]	944.0 [705.4]	**0.0001**

Data are presented as n (%) for dichotomous variables, the mean (SD) for continuous demographic variables with a normal distribution and the median [interquartile range] for those with a nonnormal distribution. The percentage was calculated based on the available data for each variable.

ALM/H^2^: appendicular lean mass adjusted to height; ALM/W: appendicular lean mass adjusted to body weight; BMD: bone mineral density; BMI: body mass index; DXA: dual-energy X-ray absorptiometry; GBP: gastric bypass; VAT: visceral adipose tissue. Osteoporosis was defined by a T-score ≤ −2.5 SD at any measured location, and low bone mass was defined by −2.5 SD < T-score ≤ −1 SD. Vitamin D deficiency was defined by a level below 30 ng/mL.

The results in bold are statistically significant (*p* < 0.05).

For comparisons of the data before and two years after surgery, a paired Student's t-test was used for variables with a normal distribution, and the Wilcoxon signed rank test was used for other continuous variables. For qualitative variables, the McNemar test was used.

### 3.3 Risk factors for the appearance of a BMD ≤ −2 SD on at least one site at 2 years after bariatric surgery

Before surgery, 618 patients (91.6 %) presented a normal DXA result (T-score > −2 SD). Two years after the surgery, 44 of them (7.1 %) presented a T-score ≤ −2 SD (Table 3).

**Table 3:** Characteristics at 2 years of the patients with a normal DXA (T-score > −2 SD) at baseline (before bariatric surgery) (n = 618).

	DXA > −2 SD at 2 years	DXA ≤ −2 SD at 2 years	*p* value
Demographical data	N = 574	N = 44	
Age	48.7 (11.0)	56.9 (8.6)	**0.0001**
Sex (women)	446 (77.7)	35 (79.5)	0.957
Weight before surgery (kg)	125.9 (22.0)	113.4 (22.0)	**0.022**
Weight after surgery (kg)	83.6 (18.4)	70.3 (15.2)	**0.0001**
BMI before surgery (kg/m2)	45.4 (6.4)	42.4 (6.4)	0.091
BMI after surgery (kg/m2)	30.1 (5.7)	26.2 (4.4)	**0.001**
Δ BMI	15.2 (5.3)	16.2 (5.7)	0.699
Diabetes	174 (30.3)	11 (25)	0.623
Cardiovascular risk factors	337 (58.7)	31 (70.5)	0.088
Tobacco	218 (38.0)	14 (31.8)	0.294
Alcohol	19 (3.3)	1 (2.3)	0.967
Vitamin D deficiency	477 (83.1)	35 (79.5)	0.674
Type of surgery			
GBP	528 (92.0)	41 (93.2)	0.745
Sleeve gastrectomy	46 (8.0)	3 (6.8)	
DXA (before surgery)			
Lean mass	58.3 (10.8)	51.5 (10.6)	**0.033**
Lean mass/total weight (%)	34.9 (5.1)	31.4 (4.9)	**0.001**
Fat mass	63.2 (13.9)	57.1 (13.0)	**0.0001**
Android fat mass/gynoid fat mass	0.77 (0.4)	0.69 (0.3)	0.722
Appendicular/central fat mass	0.8 (0.2)	0.8 (0.2)	0.12
ALM/H^2^	17.1 (2.9)	15.1 (2.8)	**0.0001**
VAT (cm^3^)	2938.3 (1489.5)	2839.5 (1405.1)	0.409

Data are presented as n (%) for dichotomous variables, the mean (SD) for continuous demographic variables with a normal distribution and the median [interquartile range] for those with a nonnormal distribution. The percentage was calculated based on the available data for each variable.

ALM/H^2^: appendicular lean mass adjusted to height; BMD: bone mineral density; BMI: body mass index; DXA: dual-energy X-ray absorptiometry; GBP: gastric bypass; VAT: visceral adipose tissue. Vitamin D deficiency was defined by a level below 30 ng/mL.

p value: a Mann-Whitney U test for variables with nonnormal distribution and Chi-squared for variables with normal distribution and logistic regression were performed to identify the variables significantly associated with the binary outcome of T-score ≤ −2 SD.

The results in bold are statistically significant (p < 0.05).

For these patients, in the univariate analysis (Table 3), the risk factors for having a T-score ≤ −2 SD at two years were an older age (*p* = 0.0001), a lower weight before and after the surgery (*p* = 0.022 and 0.0001, respectively), a lower BMI after surgery (*p* = 0.01), a lower lean and fat mass before surgery (*p* = 0.033 and 0.0001, respectively) and a lower appendicular lean mass adjusted to height (p = 0.0001).

In multivariate analysis (Table 4), the risk factors for developing a T-score ≤ −2 SD 2 years after bariatric surgery in patients with a T-score > −2 SD before surgery were age, with older patients having a higher risk (OR = 1.07, 95%CI = [1.03–1.12] and lower lean and fat mass before bariatric surgery (OR = 0.83, 95%CI = [0.77–0.91]; OR = 1.08, 95%CI = [1.02–1.15], respectively).

**Table 4:** Univariate and multivariate analysis results (Threshold ≤ −2 SD).

	Normal DXA	Abnormal DXA	Multivariate
Demographical data	N = 574	N = 44	OR (95%CI)
Age (years)	48.7 (11.0)	56.9 (8.6)	**1.07 [1.03–1.12]**
DXA (before surgery)			
Lean mass (kg)	58.58 [10.8]	55.67 [10.53]	**0.83 [0.77–0.91]**
Fat mass (kg)	63.98 [13.95]	60.01 [13.8]	**1.08 [1.02–1.15]**

Data are presented as the mean (SD) for continuous demographic variables with a normal distribution and the median [interquartile range] for those with a nonnormal distribution.

Logistic regression was performed to identify the variables significantly associated with the binary outcome of T-score ≤ −2 SD.

The results in bold are statistically significant (p < 0.05).

We found no relationship between weight change and DXA change after surgery (R^2^ = 0.04).

## 4 Discussion

In this study, a large cohort of patients, with a large predominance of women and a mean age under 50 years, who underwent bariatric surgery, representative of patients with obesity (Kanter and Caballero, 2012; Hales et al., 2020), was evaluated. The mean reduction of BMI was consistent with the data in the literature, with a BMI reduction between 13.2 and 16 kg/m^2^ after sleeve gastrectomy or GBP (Silva et al., 2019; Junquera Bañares et al., 2021). Because the amount of weight lost peaked at the 2-year follow-up and was relatively stable after this time point (O'Brien et al., 2019), studies at 2 years may be interesting in view of the weight stabilization observed. Gastric bypass (GBP) represented 91.7 % of the surgeries, and sleeve gastrectomy represented 8.3 %.

The statistically significant difference in the osteoporosis and low bone mass prevalence before and 2 years after bariatric surgery and T-score and BMD significantly lower after bariatric surgery at any site were in accordance with the literature. Previous studies have shown a decrease in BMD at the femoral neck, spine and hip (Ko et al., 2016; Paccou et al., 2022b; Krez and Stein, 2020; Jammah, 2015; von Mach et al., 2004; Pugnale et al., 2003; Mele et al., 2022). Ko et al. performed a meta-analysis (Ko et al., 2016) showing that BMD at the femoral neck decreased after bariatric surgery compared to that in nonsurgical controls, whereas BMD at the lumbar spine did not show a difference between the two groups. Bone loss following bariatric surgery is multifactorial (Krez and Stein, 2020; Mele et al., 2022), including high-turnover bone loss (Paccou et al., 2022b) and an alteration of the bone architecture with increased bone marrow adipose tissue (Ambrosi et al., 2017; Boskey and Imbert, 2017; Naveiras et al., 2009). Metabolic bone disease is due to complex interactions between signaling factors in the gut, bone, and fat tissues (Jammah, 2015). Furthermore, malabsorptive surgical techniques (sleeve gastrectomy, gastric bypass) create digestive malabsorption of calcium, vitamin D, and other nutrients (Jammah, 2015). Current management should be geared toward bone loss prevention and nutritional deficiency correction. Pharmacological treatments should be considered for high-risk patients or patients with fractures without traumatism. The percentage of bone loss is strongly correlated with the speed of weight loss (Jammah, 2015).

We found no relationship between weight loss and DXA changes after surgery. This is probably due to the low kinetics of bone modifications.

In multivariate analysis, the risk factors for the development of T-score ≤ −2 SD) at 2 years were age and lower lean and fat mass before surgery. These results were consistent with those in the literature for age (Kelsey, 1989). Concerning lean mass, our result is consistent with the literature. An involuntary loss of skeletal muscle mass was previously positively correlated with bone density and the development of fragility fractures (Hida et al., 2016; Sipilä et al., 2020), but no threshold of lean mass loss was previously described.

Our study has some limitations. Most of the bariatric surgery interventions involved GBP (91.7 %), so restrictive procedures were not evaluated. The retrospective modality did not permit us to explore the fracture events, to have more information about menopausal status, presence of endocrine diseases. We also have no specific data on medicine-related bone illness and specific treatment for osteoporosis. After surgery, all patients have a vitamin D supplementation and a calcium supplementation in case of high parathyroid hormone level and/or low calciuria and/or calcium intake <900 mg/day. DXA remains the gold standard to diagnose osteoporosis, but it is known that BMD increases with BMI (Scibora et al., 2012) and that photon penetration is reduced through soft tissues (Yu, 2014), which is why mass variation during the follow-up could have influenced the DXA values, with an underestimation of bone fragility for patients with elevated fat mass.

In a previous study (Halin et al., 2022), we found similar results with a CT osteoporosis study. There was a higher prevalence of bone fragility 2 years after bariatric surgery than before. Unfortunately, the populations of these 2 studies were similar but not identical, and thus we cannot compare the 2 modalities of osteoporosis screening. The correlation between CT and DXA, which was previously studied in patients with obesity, is positive but poor (Halin et al., 2022). This lack of relationship could be explained by the difference in measurements between the 2 exams. CT excludes cortical bone, is less influenced by body fat mass and is more representative of real bone mass than DXA (Halin et al., 2022). In the literature, Yu et al. also found discordant results between DXA and QCT bone density (Yu et al., 2014).

In conclusion, there is a higher prevalence of osteoporosis and low bone mass after bariatric surgery, and these results should encourage practitioners to perform bone screening before and after this surgery to diagnose osteoporosis before fracture involvement, according to the recommendations (Paccou et al., 2022a; Mechanick et al., 2013), especially in older patients with a lower lean and fat mass at baseline. All of the risk factors for development of a BMD ≤ −2 SD at 2 years (indication of anti-osteoporosis treatment for menopausal women and men ≥50 years old) were preoperative characteristics. Longitudinal follow-up may be warranted to precisely determine the kinetics of bone loss after bariatric surgery and to establish thresholds for screening or therapeutic interventions.

## Funding

None.

## CRediT authorship contribution statement

**Marion Halin:** Writing – review & editing, Writing – original draft, Validation, Methodology, Investigation, Formal analysis, Data curation, Conceptualization. **Edem Allado:** Validation, Methodology, Formal analysis, Conceptualization. **Laurent Brunaud:** Writing – review & editing, Validation, Supervision, Methodology, Formal analysis, Conceptualization. **Claire Nomine-Criqui:** Writing – review & editing, Validation, Methodology. **Eliane Albuisson:** Writing – review & editing, Validation, Supervision, Methodology, Formal analysis, Conceptualization. **Isabelle Chary-Valckenaere:** Writing – review & editing, Validation, Supervision, Methodology, Conceptualization. **Didier Quilliot:** Writing – review & editing, Validation, Supervision, Methodology, Conceptualization. **Damien Loeuille:** Writing – review & editing, Validation, Supervision, Methodology, Conceptualization.

## Declaration of competing interest

Marine Fauny, Marion Halin, Edem Allado, Laurent Brunaud, Claire Nomine-Criqui, Eliane Albuisson, Isabelle Chary-Valckenaere, Didier Quilliot, and Damien Loeuille declare that they have no conflict of interest.

## Data Availability

Data will be made available on request.

## References

[bb0005] Ambrosi T.H., Scialdone A., Graja A. (2017 Jun 1). Adipocyte accumulation in the bone marrow during obesity and aging impairs stem cell-based hematopoietic and bone regeneration. Cell Stem Cell.

[bb0010] Apovian C.M. (2016 Jun). Obesity: definition, comorbidities, causes, and burden. Am. J. Manag. Care.

[bb0015] Axelsson K.F., Werling M., Eliasson B. (2018 Dec). Fracture risk after gastric bypass surgery: a retrospective cohort study. J. Bone Miner. Res..

[bb0020] Borisenko O., Colpan Z., Dillemans B., Funch-Jensen P., Hedenbro J., Ahmed A.R. (2015 Aug). Clinical indications, utilization, and funding of bariatric surgery in Europe. Obes. Surg..

[bb0025] Boskey A.L., Imbert L. (2017 Dec). Bone quality changes associated with aging and disease: a review. Ann. N. Y. Acad. Sci..

[bb0030] Dimai H.P. (Nov 2017). Use of dual-energy X-ray absorptiometry (DXA) for diagnosis and fracture risk assessment; WHO-criteria, T- and Z-score, and reference databases. Bone.

[bb0035] Geoffroy M., Charlot-Lambrecht I., Chrusciel J. (2019 Jun). Impact of bariatric surgery on bone mineral density: observational study of 110 patients followed up in a specialized Center for the Treatment of obesity in France. Obes. Surg..

[bb0040] Gonnelli S., Caffarelli C., Nuti R. (2014 Jan). Obesity and fracture risk. Clin. Cases Miner. Bone Metab..

[bb0045] Hales C.M., Carroll M.D., Fryar C.D., Ogden C.L. (2020 Feb). Prevalence of obesity and severe obesity among adults: United States, 2017-2018. NCHS Data Brief.

[bb0050] Halin M., Allado E., Albuisson E. (2022 Jan). Prevalence of osteoporosis assessed by DXA and/or CT in severe obese patients. J. Clin. Med..

[bb0055] Hida T., Shimokata H., Sakai Y. (2016 Nov). Sarcopenia and sarcopenic leg as potential risk factors for acute osteoporotic vertebral fracture among older women. Eur. Spine J..

[bb0060] Hofsø D., Hillestad T.O.W., Halvorsen E., Fatima F., Johnson L.K., Lindberg M. (2020). Bone mineral density and turnover after sleeve gastrectomy and gastric bypass: a randomized controlled trial (Oseberg). J. Clin. Endocrinol. Metab..

[bb0065] Jammah A.A. (2015). Endocrine and metabolic complications after bariatric surgery. Saudi J. Gastroenterol..

[bb0070] Junquera Bañares S., Ramírez Real L., Camuñas Segovia J., Martín García-Almenta M., Llanos Egüez K., Álvarez Hernández J. (2021). Evaluation of quality of life, weight loss and evolution of comorbidities at 6 years after bariatric surgery. Endocrinol Diabetes Nutr (Engl Ed)..

[bb0075] Kanter R., Caballero B. (2012 Jul 1). Global gender disparities in obesity: a review. Adv. Nutr..

[bb0080] Kelsey J.L. (1989). Risk factors for osteoporosis and associated fractures. Public Health Rep..

[bb0085] Ko B.-J., Myung S.K., Cho K.-H. (2016 Jul). Relationship between bariatric surgery and bone mineral density: a Meta-analysis. Obes. Surg..

[bb0090] Krez A.N., Stein E.M. (2020 Jun). The skeletal consequences of bariatric surgery. Curr. Osteoporos. Rep..

[bb0095] Lane J.M., Russell L., Khan S.N. (2000 Mar). Osteoporosis. Clin. Orthop. Relat. Res..

[bb0100] Lespessailles E., Paccou J., Javier R.-M., Thomas T., Cortet B. (2019 Oct 1). GRIO scientific committee. Obesity, bariatric surgery, and fractures. J. Clin. Endocrinol. Metab..

[bb0105] Lespessailles E., Hammoud E., Toumi H., Ibrahim-Nasser N. (2019 Mar 28). Consequences of bariatric surgery on outcomes in rheumatic diseases. Arthritis Res. Ther..

[bb0110] Lu C.-W., Chang Y.-K., Chang H.-H. (2015 Dec). Fracture risk after bariatric surgery: a 12-year Nationwide cohort study. Medicine (Baltimore).

[bb0115] Lupoli R., Lembo E., Saldalamacchia G., Avola C.K., Angrisani L., Capaldo B. (2017 Nov 15). Bariatric surgery and long-term nutritional issues. World J. Diabetes.

[bb0120] Mechanick JI, Youdim A, Jones DB, et al. Clinical practice guidelines for the perioperative nutritional, metabolic, and nonsurgical support of the bariatric surgery patient--2013 update: cosponsored by American Association of Clinical Endocrinologists, The Obesity Society, and American Society for Metabolic & Bariatric Surgery. Obesity (Silver Spring) 2013 Mar;21 Suppl 1:S1–27. 10.1002/oby.20461 PMC4142593 23529939

[bb0125] Mele C., Caputo M., Ferrero A. (2022). Bone response to weight loss following bariatric surgery. Front Endocrinol (Lausanne)..

[bb0130] Murray T.É., Williams D., Lee M.J. (2017). Osteoporosis, obesity, and sarcopenia on abdominal CT: a review of epidemiology, diagnostic criteria, and management strategies for the reporting radiologist. Abdom Radiol (NY)..

[bb0135] Naveiras O., Nardi V., Wenzel P.L., Hauschka P.V., Fahey F., Daley G.Q. (2009 Jul 9). Bone-marrow adipocytes as negative regulators of the haematopoietic microenvironment. Nature.

[bb0140] O’Brien P.E., Hindle A., Brennan L. (2019 Jan). Long-term outcomes after bariatric surgery: a systematic review and Meta-analysis of weight loss at 10 or more years for all bariatric procedures and a single-Centre review of 20-year outcomes after adjustable gastric banding. Obes. Surg..

[bb0145] Paccou J., Genser L., Lespessailles É., Bertin É., Javier R.M., Duclos M. (2022 Nov). French recommendations on the prevention and treatment of osteoporosis secondary to bariatric surgery. Joint Bone Spine.

[bb0150] Paccou J., Caiazzo R., Lespessailles E., Cortet B. (2022 May). Bariatric surgery and osteoporosis. Calcif. Tissue Int..

[bb0155] Pugnale N., Giusti V., Suter M. (2003 Jan). Bone metabolism and risk of secondary hyperparathyroidism 12 months after gastric banding in obese pre-menopausal women. Int. J. Obes. Relat. Metab. Disord..

[bb0160] Sarafrazi N. Osteoporosis or Low Bone Mass in Older Adults: United States, 2017-2018 [Internet]. National Center for Health Statistics; 2021 Mar [cited 2023 Apr 14]. Available from:https://stacks.cdc.gov/view/cdc/103477. 34029181

[bb0165] Scibora L.M., Ikramuddin S., Buchwald H., Petit M.A. (2012 Apr). Examining the link between bariatric surgery, bone loss, and osteoporosis: a review of bone density studies. Obes. Surg..

[bb0170] Silva L.B., Oliveira B.M.P.M., Correia F. (2019 Jun). Evolution of body composition of obese patients undergoing bariatric surgery. Clin Nutr ESPEN..

[bb0175] Sipilä S., Törmäkangas T., Sillanpää E. (2020 Jun). Muscle and bone mass in middle-aged women: role of menopausal status and physical activity. J. Cachexia. Sarcopenia Muscle.

[bb0180] Tencerova M., Frost M., Figeac F. (2019 May 14). Obesity-associated Hypermetabolism and accelerated senescence of bone marrow stromal stem cells suggest a potential mechanism for bone fragility. Cell Rep..

[bb0185] von Mach M.A., Stoeckli R., Bilz S., Kraenzlin M., Langer I., Keller U. (2004 Jul). Changes in bone mineral content after surgical treatment of morbid obesity. Metabolism.

[bb0190] WHO Obesity: preventing and managing the global epidemic. Report of a WHO consultation. World Health Organ. Tech. Rep. Ser. 2000;894:i–xii, 1–253. 11234459

[bb0195] (1994). Assessment of fracture risk and its application to screening for postmenopausal osteoporosis. Report of a WHO study group. World Health Organ. Tech. Rep. Ser..

[bb0200] Yu E.W. (2014 Jul). Bone metabolism after bariatric surgery. J. Bone Miner. Res..

[bb0205] Yu E.W., Bouxsein M.L., Roy A.E. (2014 Mar). Bone loss after bariatric surgery: discordant results between DXA and QCT bone density. J. Bone Miner. Res..

